# IL-6-driven FasL promotes NF-κBp65/PUMA-mediated apoptosis in portal hypertensive gastropathy

**DOI:** 10.1038/s41419-019-1954-x

**Published:** 2019-10-03

**Authors:** Siwei Tan, Minyi Xu, Bilun Ke, Yu Lu, Huiling Liu, Jie Jiang, Bin Wu

**Affiliations:** 1 Department of Gastroenterology, The Third Affiliated Hospital of Sun Yat-Sen University, Guangzhou, 510630 Guangzhou China; 2 Guangdong Provincial Key Laboratory of Liver Disease Research, 510630 Guangzhou, China

**Keywords:** Apoptosis, Cell death and immune response

## Abstract

Mucosal epithelial apoptosis with non-specific inflammation is an essential pathological characteristic in portal hypertensive gastropathy (PHG). However, whether a coordinated crosstalk between myeloid cells and epithelial cells involved in PHG remains unclear. IL-6, which is induced in the mucosa of PHG patients and mice, promotes FasL production via enhancing NF-κBp65 activation in myeloid cells, while blockage of IL-6 signaling by Tocilizumab or deletion of *NF-κBp65* in myeloid cells attenuates the inflammatory response and Fas/FasL-mediated epithelial apoptosis in PHG. IL-6-driven FasL from myeloid cells combines with epithelial Fas receptor to encourage NF-κBp65/PUMA-mediated epithelial apoptosis in PHG, and inhibition of NF-κBp65 or knockout of *PUMA* alleviates Fas/FasL-mediated epithelial apoptosis in PHG. These results indicate that IL-6 drives FasL generation via NF-κBp65 in myeloid cells to promote Fas/NF-κBp65/PUMA-mediated epithelial apoptosis in PHG, and this coordinated crosstalk between myeloid cells and epithelial cells may provide a potential therapeutic target for PHG.

## Introduction

Portal hypertensive gastropathy (PHG) occurs as the most common gastric mucosal injury among patients afflicted with cirrhotic or non-cirrhotic portal hypertension^1–4^. Accumulating studies suggest that portal hypertension, prostaglandins (PGs), tumor necrosis factor-α (TNF-α) and excessive nitric oxide (NO), oxygen free radicals and lipid peroxidation are implicated in the pathogenesis of PHG^2,5,6^. We and others have identified that gastric epithelial apoptosis plays a crucial role in the formation of PHG^7,8^. Apoptosis has been implicated in a series of biochemical steps as well as tissue damage under a number of pathological conditions^9^. Three classic signaling networks, namely, TNF-α/TNF receptor 1 (TNFR1), Fas ligand (FasL)/Fas and TNF-related apoptosis-inducing ligand (TRAIL)/death receptor 4 (DR4, also called TRAIL-R1)/death receptor 5 (DR5, also called TRAIL-R2), could conserve the modulators that mediate caspase-dependent cellular apoptosis through regulating the signaling pathways such as nuclear factor-κB (NF-κB), etc^7,10–12^. Our previous researches showed that portal hypertension triggered TNF-α, Fas/FasL levels and endoplasmic reticulum (ER) stress/p53-upregulated modulator of apoptosis (PUMA)-induced mucosal epithelial apoptosis in PHG^7,8^. Fas, following FasL engagement, leads to the recruitment of Fas-associated proteins having death domains and the initiator procaspase-8 via its death effector domain into a death-inducing signaling complex (DISC) to generate executioner caspase cleavage, which leads to cell death^11,13,14^. Although the function of TNF-α in gastric epithelial apoptosis has been extensively illustrated, the Fas/FasL system in PHG is still poorly understood.

NF-κB, a ubiquitous transcription factor, is a complex of dimeric subunits that belongs to the NF-κB/Rel family: NF-κB1 (p50 and its precursor p105), NF-κB2 (p52 and its precursor p100), p65 (RelA), RelB, and c-Rel^15^. As the main functional element, NF-κBp65 was revealed to be responsible for mediating inflammation, development, injury, apoptosis and proliferation in various physiological and pathological courses^8,16,17^. Previous reports have established that NF-κBp65 activation involved in TNF-α or IL-6 signaling in the development of colitis, breast cancer, colorectal cancer, lymphoma cells^15,18,19^. IL-6, as a pleiotropic cytokine, is secreted by and elicits responses in a wide range of cells. It not only regulates immunity through effects on the generation, recruitment, secretion, and transformation of neutrophils, macrophages and dendritic cells but also stimulates the production of antibodies by B cells, contributing to shaping the T cells balance via regulating the signal transducer and activator of transcription 3 (STAT3) and NF-κB pathways^20,21^.

In the current study, we found that IL-6 was induced in the mucosa of PHG, and that IL-6 promoted FasL production in myeloid cells via NF-κBp65 activation, which could bind to the epithelial Fas receptor to encourage NF-κBp65/PUMA-mediated epithelial apoptosis in PHG. Furthermore, inhibition of IL-6 signaling or specific deletion of *NF-κBp65* in myeloid cells attenuated the inflammatory response and Fas/FasL-mediated epithelial apoptosis in PHG. These results indicated that IL-6 drives FasL production via NF-κBp65 in myeloid cells to promote Fas/NF-κBp65/PUMA-mediated epithelial apoptosis in PHG.

## Results

### IL-6 involved in PHG

**Fig. 1:**
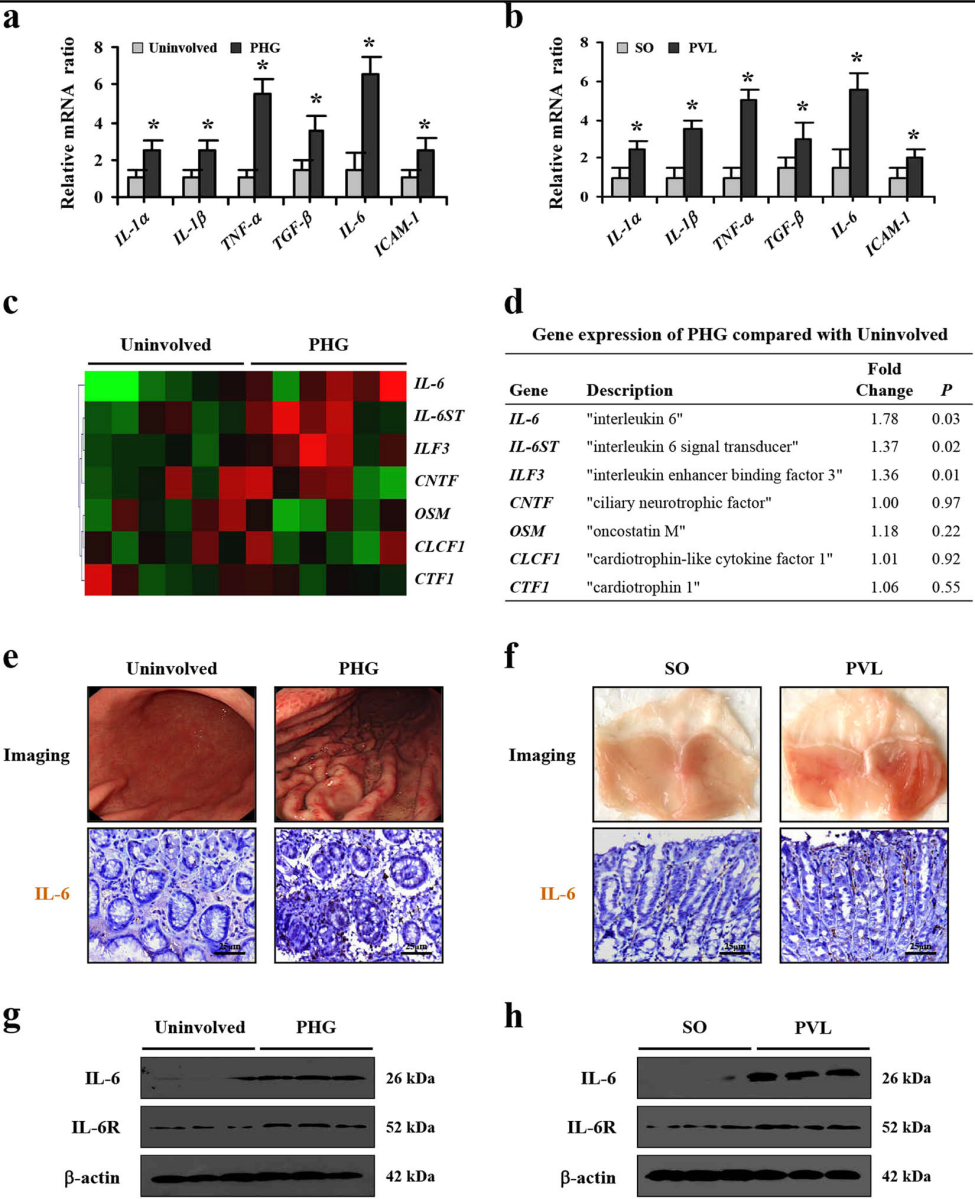
IL-6 involved in PHG. **a**, **b** Expressions of indicated inflammatory cytokines in the related gastric mucosa were analyzed. *β-actin* was used as an internal control. *n* = 6 in each group, values are presented as mean ± SEM. Bonferroni’s comparison post hoc test. *P < 0.05 versus uninvolved tissues or SO (sham operation) mice, respectively. **c** Two-dimensional hierarchical clustering results for the gene of IL-6 superfamily members between PHG patients and healthy volunteers (*n* = 6 per group). **d** The fold changes and *P* value of the indicated mRNA levels in PHG tissues relative to normal (uninvolved) tissues from microarray experiment were represented. **e** Endoscopic imaging and IL-6 immunohistochemistry (IHC) staining of uninvolved normal gastric mucosal tissue and gastropathic mucosal tissue from PHG patient were presented (brown, × 400, *n* = 6 per group). **f** IL-6 staining and gastric imaging in the indicated sections from SO and PVL mice (brown, × 400, *n* = 6 per group). **g**, **h** IL-6 and IL-6R levels in the indicated gastric mucosa were determined by western blotting in three pairs of different representative specimens. β-actin was used as the loading control. *n* = 6 per group

Previous studies have demonstrated that various elements, such as prostaglandins, TNF-α, oxygen free radicals and TGF-β, participated in the pathogenesis of PHG^8,22,23^. Based on the importance of the inflammatory response in mucosa injury, we quantified several inflammatory factors in the stomach by real-time PCR. The levels of *IL-1α*, *IL-1β*, *TNF-α*, *TGF-β*, *IL-6*, and *ICAM-1* were markedly increased in PHG mucosal tissues (Fig. 1a). Using the partial portal vein ligation (PVL)-treated mouse model, the levels of the above-mentioned mediators were also increased (Fig. 1b). The expression of *IL-6* was the highest compared with that of other factors in PHG both from patients and mice. A gene microanalysis was performed to screen for the genes expression alteration of the IL-6 superfamily members in the blood of both PHG patients and healthy volunteers, and we found that IL-6 and its signal transducer in PHG samples showed higher expression than in normal samples (Fig. 1c, d). Histopathological detection presented a loss of gastric preserved architecture in PHG and the expression of IL-6 was remarkably higher in the tissues of PHG patients and PVL mice compared with that in their normal groups, and IL-6 was nearly located in the gastric mesenchymal cells (Fig. 1e, f). Western blotting also revealed a similar situation (Fig. 1g, h). Based on these, we conclude that IL-6 involved in PHG.

### Inhibition of IL-6 signaling attenuated the inflammatory response and Fas-mediated epithelial apoptosis in PHG

**Fig. 2:**
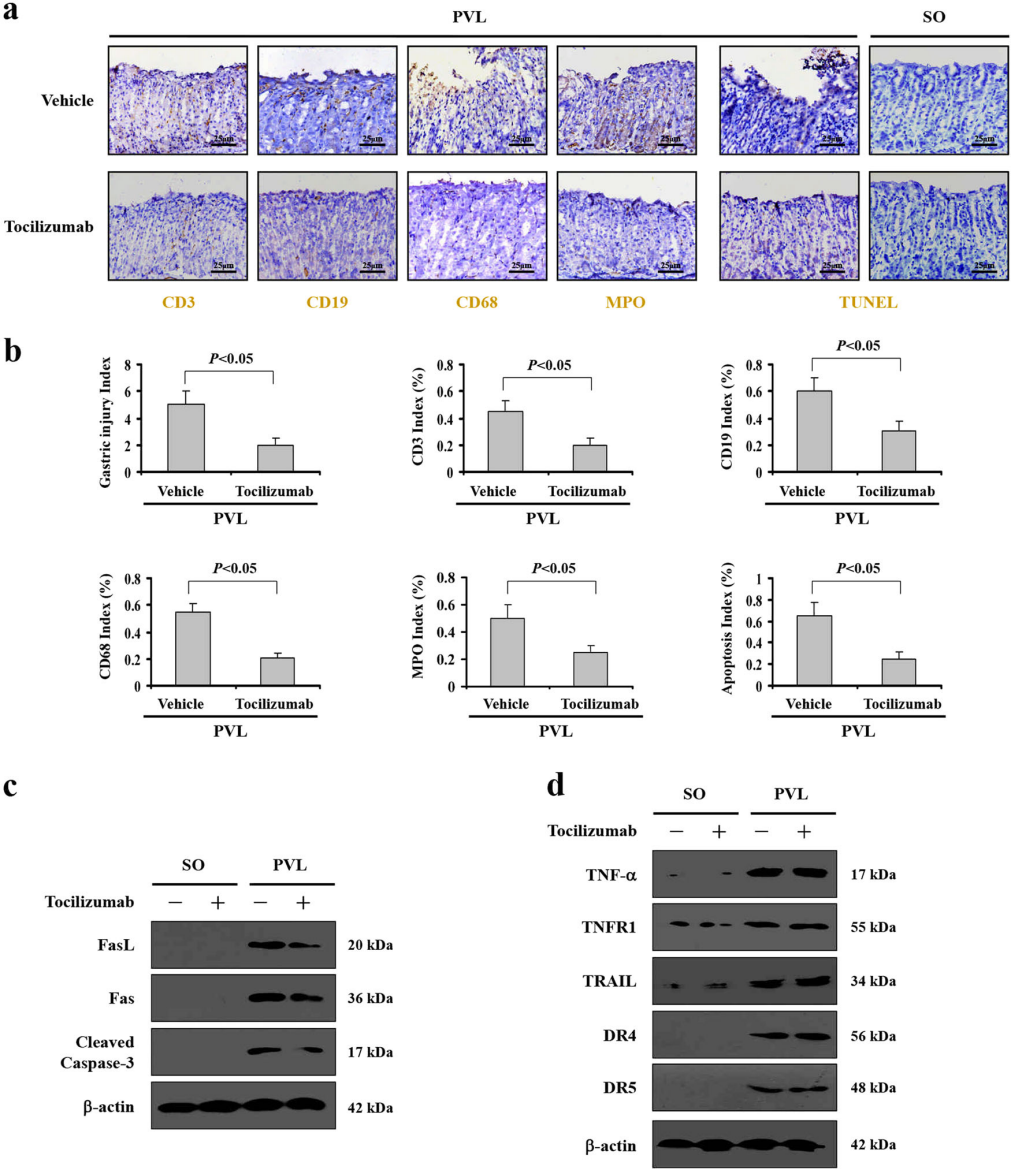
Inhibition of IL-6 signaling attenuated inflammatory response and Fas-mediated epithelial apoptosis in PHG. **a** The indicated inflammatory cell staining and mucosal TUNEL staining in the tissues were analyzed (brown, × 400, *n* = 6 per group). **b** The gastric injury index (left panel), CD68 index (macrophage), CD19 index (B cell), CD3 index (T cell), MPO index (neutrophil), and the apoptosis index (TUNEL staining) analyzed from (**a**). *n* = 6 in each group, values are presented as mean ± SEM. **c** Gastric mucosal Fas, FasL and cleaved caspase-3 expression were evaluated by western blotting. β-actin was used as the loading control. *n* = 6 per group. **d** Western blotting analyses revealed that the levels of TNF-α/TNFR1 and TRAIL/DR4/DR5 in PVL mice were not affected by Tocilizumab administration. β-actin was used as the loading control. *n* = 6 per group

Based on the involvement of IL-6 in PHG, the selective IL-6 receptor (IL-6R) inhibitor Tocilizumab was used. Tocilizumab notably alleviated macrophage (CD68), B cell (CD19), T cell (CD3) and neutrophil (myeloperoxidase, MPO) infiltration, as well as gastric injury and mucosal apoptosis, in the tissues of PVL-treated mice (Fig. 2a, b). Apoptosis is mediated through a series of biochemical steps, and three common signaling networks, TNF-α/TNFR1, Fas/FasL, and TRAIL/DR4/DR5, could contribute to the induction of apoptosis^7,11^. In SO (sham operation) mice, small amounts of gastric mucosal TNF-α/TNFR1, Fas/FasL, and TRAIL/DR4/DR5 were detected (Fig. 2c, d). The amounts of gastric mucosal TNF-α/TNFR1, Fas/FasL, and TRAIL/DR4/DR5 were significantly increased in PVL mice compared with the amounts in SO groups. While, inhibition of IL-6R with Tocilizumab suppressed Fas/FasL levels without affecting the TNF-α/TNFR1 and TRAIL/DR4/DR5 induction, although it did alleviate mucosal epithelial apoptosis in PVL-treated mice (Fig. 2c, d). These results suggested inhibition of the IL-6 network attenuated the inflammatory response and Fas/FasL-mediated apoptosis in PHG.

### IL-6 drove epithelial apoptosis via upregulating myeloid FasL

**Fig. 3:**
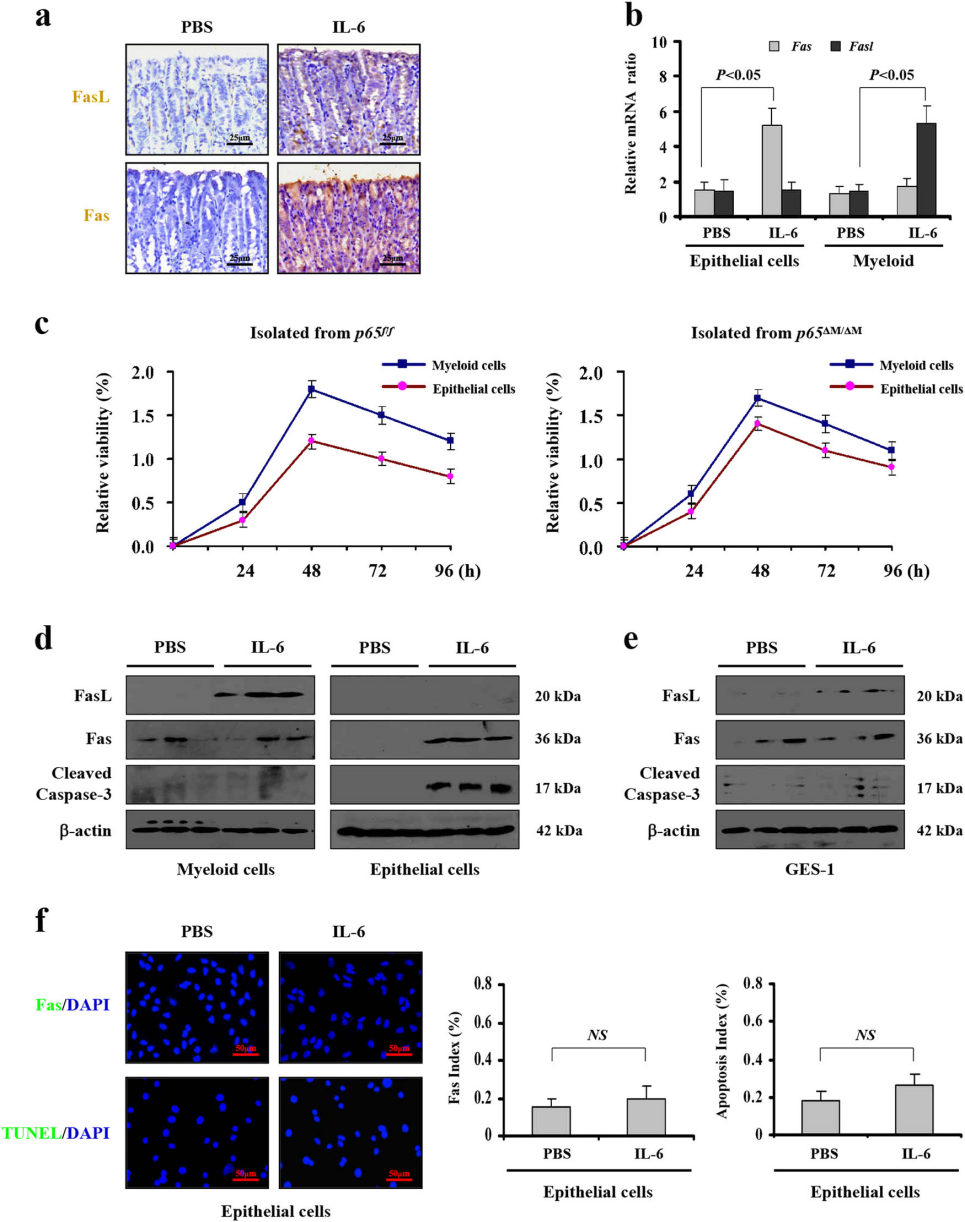
IL-6 drove epithelial apoptosis via upregulating myeloid FasL. **a** Immunohistochemical staining showed that IL-6 administration upregulated the expression of Fas and FasL in mice gastric mucosa (*n* = 6 per group). **b** *Fas* and *Fasl* mRNA from isolated primary epithelial and myeloid cells analyzed by PCR. *β-actin* was used as an internal control. *n* = 6 in each group, values are presented as mean ± SEM. Bonferroni’s comparison post hoc test. **c** The viability of primary myeloid cells and epithelial cells isolated from *p65*^*f/f*^ (floxed *p65*) mice or *p65*^ΔM/ΔM^ (myeloid cells specific *NF-κBp65* deletion) mice were assayed by the Cell Counting Kit-8 (CCK-8) analysis, respectively. *n* = 6 per group. **d** FasL, but not Fas and cleaved caspase-3, was induced in primary myeloid cells of IL-6-treated mice (left panel). Fas and cleaved caspase-3, but not FasL, were induced in primary epithelial cells of IL-6-treated mice (right panel). *n* = 6 in each group, values are presented as mean ± SEM. **e** Expressions of Fas, FasL and cleaved caspase-3 in GES-1 cells analyzed by western blotting. β-actin was used as the loading control. *n* = 6 in each group, values are presented as mean ± SEM. **f** Immunofluorescence staining of Fas (green) and TUNEL staining indicated IL-6 could not promote primary epithelial cells apoptosis. Cell nuclei (blue) were counterstained by DAPI (× 800). The Fas index and the apoptotic index were also represented. *n* = 6 in each group, values are presented as mean ± SEM. *NS*, no significance

To investigate the mechanism of IL-6-driven mucosal epithelial apoptosis via the Fas/FasL signaling, intravenously injected recombinant murine IL-6 mice were adopted. Histopathological analysis revealed IL-6 treatment induced Fas and FasL expression compared with PBS (vehicle) administration (Fig. 3a). By analyzing the mRNA in primary epithelial and myeloid cells isolated from the IL-6- or PBS-treated mice, we found that IL-6 induced *FasL* almost in myeloid cells, while *Fas* was increased in epithelial cells (Fig. 3b). The primary viability by the Cell Counting Kit-8 (CCK-8) analysis represented that they obtained the highest viability after being cultured for 48 h without any treatments (Fig. 3c). The amount of FasL protein was increased in the myeloid fractions, while Fas protein was mainly upregulated in the epithelial elements from the gastric mucosa after IL-6 treatment (Fig. 3d). Furthermore, IL-6 treatment did not promote the expression of Fas and the apoptosis both in the primary epithelial cells and the gastric epithelial cell lines (GES-1) (Fig. 3e, f and Supplementary Fig. 1), revealing that IL-6 could not promote Fas upregulation and apoptosis of gastric epithelial cells directly, and IL-6 enhanced Fas-mediated epithelial apoptosis via upregulating myeloid FasL production in PHG.

### IL-6 upregulated FasL levels via NF-κBp65 in myeloid cells in PHG

**Fig. 4:**
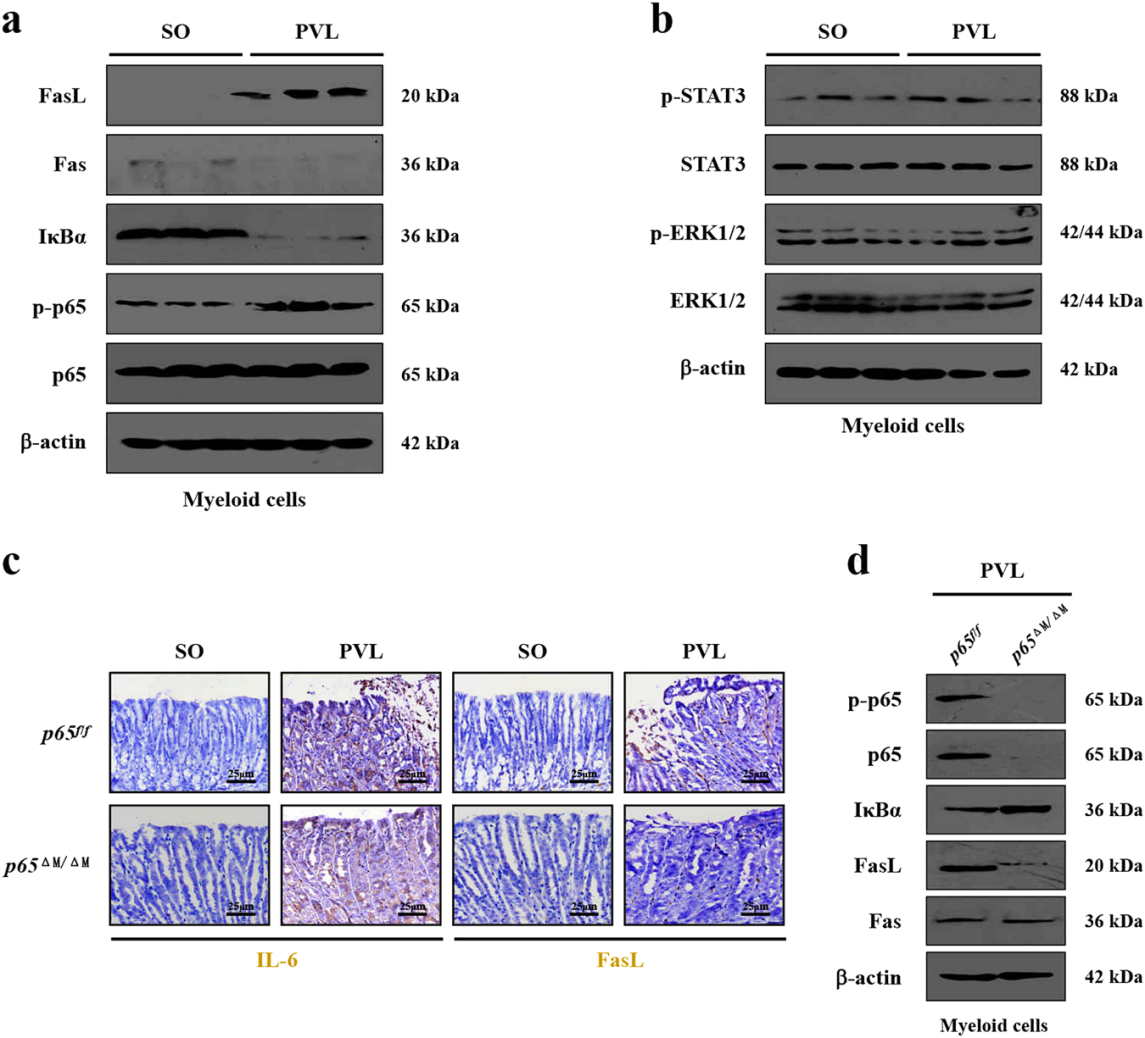
IL-6 upregulated FasL levels via NF-κBp65 in myeloid cells in PHG. **a** FasL and NF-κBp65 phosphorylation (NF-κBp-p65, as p-p65), rather than Fas, were induced in primary myeloid cells isolated from PVL-treated mice. β-actin was used as the loading control. *n* = 6 per group. **b** The activity of STAT3 and ERK1/2 were detected in primary myeloid cells isolated from SO (sham operation)- and PVL-treated mice. β-actin was used as the loading control. *n* = 6 per group. **c** *NF-κBp65* deficiency in myeloid cells downregulated the expression of FasL, without affecting the level of IL-6 in the gastric mucosa of PVL mice (brown, × 400, *n* = 6 per group). **d** The levels of NF-κBp-p65, NF-κBp65, IκBα, FasL and Fas in the primary myeloid cells dissociated from PVL-treated mice were determined. *p65*^*f/f*^, floxed *p65* mice. *p65*^ΔM/ΔM^, myeloid cells specific *NF-κBp65* deletion mice. β-actin was used as the loading control. *n* = 6 per group

By utilizing primary myeloid cells, we found that FasL levels, together with the NF-κBp65 phosphorylation (NF-κBp-p65), were increased in primary myeloid cells isolated from the PVL-treated mice compared with those from SO mice (Fig. 4a), while the activation of STAT3 and ERK1/2 was not affected in PVL-treated mice (Fig. 4b). Fas in primary myeloid cells showed no significant difference between SO mice and PVL mice (Fig. 4a), indicating that IL-6 may not promote myeloid cells apoptosis via Fas signaling, which is consistent with our previous research showing that gastric epithelial apoptosis rather than other cells apoptosis plays a specific role in the pathogenesis of PHG. PVL-treated *p65*^ΔM/ΔM^ (myeloid cells specific *NF-κBp65* deletion) mice and *p65*^*f/f*^ (floxed *p65*) mice were used and indicated that targeted deletion of *NF-κBp65* in myeloid cells ameliorated the FasL deposition in PVL-treated mice without influencing the status of IL-6, although there was no obvious distinction between *p65*^*f/f*^ and *p65*^ΔM/ΔM^ mice from the control group (Fig. 4c). The levels of NF-κBp-p65 and FasL were abolished in primary myeloid cells isolated from PVL-treated *p65*^ΔM/ΔM^ mice, in contrast to the levels in *p65*^*f/f*^ mice (Fig. 4d). By using RAW 264.7 cells in vitro, we found that IL-6 induced NF-κBp65 phosphorylation and FasL production rather than Fas, while Bay11708 (BAY), which could inhibit the activation of NF-κBp65, blocked FasL secretion concomitantly with NF-κBp65 activation (Supplementary Fig. 2a–d). Transfection of *NF-κBp65* in RAW 264.7 cells induced FasL mRNA and protein levels following IL-6 treatment (Supplementary Fig. 2e, f). These results suggested IL-6 upregulated FasL in myeloid cells via NF-κBp65 in PHG.

### IL-6-driven FasL contributed to epithelial apoptosis via Fas in PHG

**Fig. 5:**
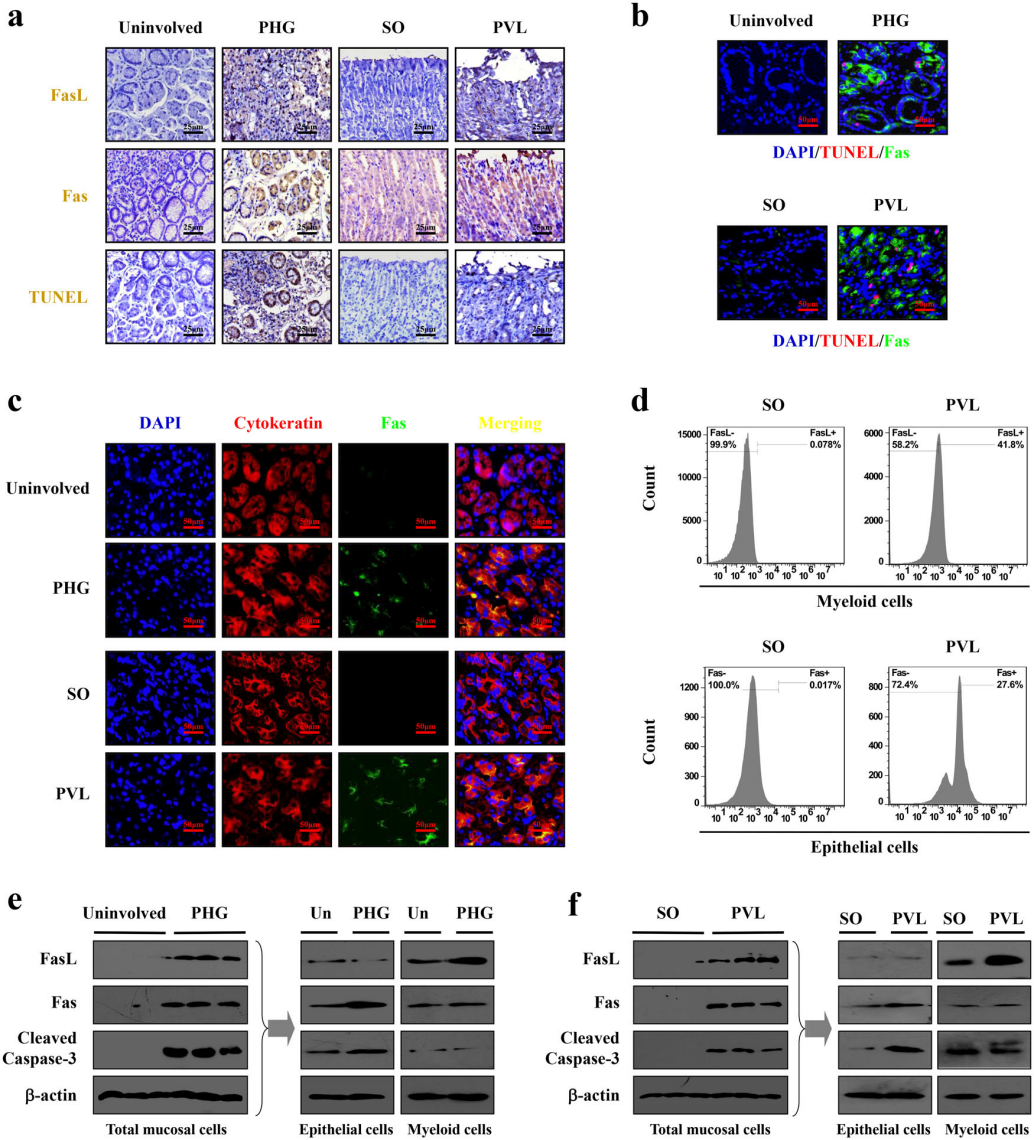
IL-6-driven FasL contributed to epithelial apoptosis via Fas in PHG. **a** Fas, FasL staining and TUNEL staining from the indicated gastric tissues were revealed (brown, × 400, *n* = 6 per group). **b** Double staining of Fas (green) and TUNEL (red) indicated that Fas-mediated apoptosis contributed to PHG, nuclei (blue) were counterstained with DAPI (× 800, *n* = 6 per group). **c** Co-staining of Fas (green) and cytokeratin (red) demonstrated that Fas mainly located in the epithelial cells of both PHG patients and mice, nuclei (blue) were counterstained with DAPI (× 800, *n* = 6 per group). **d** Flow cytometric analysis of primary myeloid and epithelial cells isolated from SO (sham operation)- and PVL-treated mice with anti-Fas or anti-FasL antibodies (*n* = 6 per group). **e** Western blotting showed that Fas, FasL and cleaved caspase-3 were enhanced in total mucosa of PHG sections (left panel). The levels of Fas, FasL and cleaved caspase-3 in the primary myeloid and epithelial cells dissociated from PHG patients and healthy volunteers (as Un, uninvolved) were determined (right panel). β-actin was used as the loading control. *n* = 6 per group. **f** Fas, FasL and cleaved caspase-3 were enhanced in PVL-treated sections. The expressions of Fas, FasL and cleaved caspase-3 in the primary myeloid and epithelial cells dissociated from SO- and PVL-treated mice were determined (right panel). β-actin was used as the loading control. *n* = 6 per group

Fas and FasL, in parallel with the increased apoptotic cells, were induced in the tissues of PHG compared with that of the control group (Fig. 5a). Double staining of Fas and TUNEL demonstrated Fas was involved in mucosal apoptosis in PHG (Fig. 5b). Labeling epithelial cells with cytokeratin revealed Fas signaling was mainly localized in the mucosal epithelial cells (Fig. 5c). Western blotting indicated that Fas and FasL, accompanied by cleaved caspase-3, were increased in both PHG patients and mice (Fig. 5e, f). By analyzing primary epithelial and myeloid cells dissociated from the related sections, we found that in both human and mouse PHG tissues, the levels of Fas and cleaved caspase-3 were increased in primary epithelial cells, while FasL was increased in myeloid cells (Fig. 5e, f). Flow cytometric analysis of primary myeloid and epithelial cells isolated from SO- and PVL-treated mice represented a similar condition (41.8% of the primary myeloid cells were FasL-positive in PVL-treated mice, while 27.6% of the primary epithelial cells were Fas-positive in PVL-treated mice) (Fig. 5d). The data indicated IL-6-driven FasL accelerated gastric epithelial apoptosis via Fas in PHG.

### *NF-κBp65* deficiency in gastric myeloid cells attenuated Fas-mediated epithelial apoptosis in PHG

**Fig. 6:**
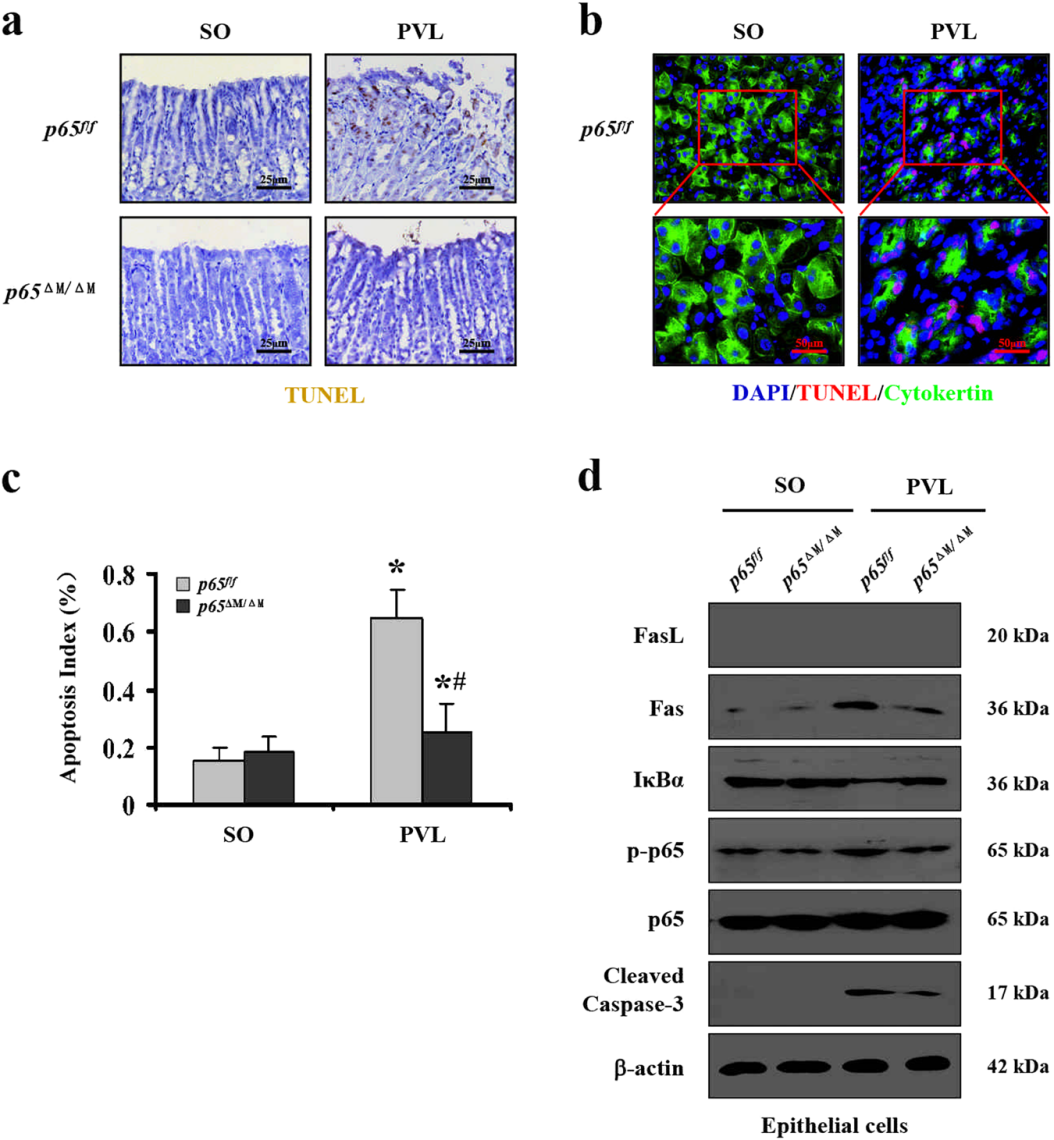
*NF-κBp65* deficiency in gastric myeloid cells attenuated Fas-mediated epithelial apoptosis in PHG. **a** Gastric mucosal apoptosis was depressed in PVL-treated *p65*^ΔM/ΔM^ (myeloid cells specific *NF-κBp65* deletion) mice compared with *p65*^*f/f*^ (floxed *p65*) mice (TUNEL staining, × 400, *n* = 6 per group). **b** Double staining of cytokeratin (green) and TUNEL (apoptotic cells, red) indicated that gastric apoptotic cells mainly located in the epithelial cells of the gastric mucosa from PVL mice (upper, × 400; lower panel, × 800, *n* *=* 6 per group). **c** The apoptotic index from TUNEL staining was presented. *n* = 6 in each group, values are presented as mean ± SEM. *P < 0.05 versus SO (sham operation) mice, ^#^*P* < 0.05 versus PVL-treated *p65*^*f/f*^ mice. **d** Western blotting analysis demonstrated that deletion of *NF-κBp65* in myeloid cells blocked Fas, cleaved caspase-3 levels and NF-κBp65 phosphorylation in the gastric epithelial cells from PVL-treated mice. β-actin was used as the loading control. *n* = 6 per group

The PHG models established by adopting *p65*^*f/f*^ and *p65*^ΔM/ΔM^ mice were used to further investigate whether Fas-mediated epithelial apoptosis responded to myeloid cells in PHG. Concurrently, few TUNEL positive cells were detected in the gastric mucosa of SO mice for both of *p65*^*f/f*^ and *p65*^ΔM/ΔM^ mice. However, apoptosis was attenuated in PVL-treated *p65*^ΔM/ΔM^ mice compared with that in PVL-treated *p65*^*f/f*^ mice (Fig. 6a, c). By utilizing the epithelial marker cytokeratin, we found the apoptotic cells were mainly localized in epithelial cells (Fig. 6b). Primary epithelial cells isolated from *p65*^*f/f*^ and *p65*^ΔM/ΔM^ mice confirmed that *NF-κBp65* deficiency in gastric myeloid cells evidently inhibited the levels of Fas, the activation of caspase-3 and the phosphorylation of NF-κBp65 in epithelial cells from PVL mice, although few FasL was detected in the epithelial cells in both *p65*^*f/f*^ and *p65*^ΔM/ΔM^ mice (Fig. 6d). These data demonstrated that deletion of *NF-κBp65* in myeloid cells attenuated Fas-mediated epithelial apoptosis in PHG.

### Fas/FasL promotes epithelial apoptosis via NF-κBp65 in PHG

**Fig. 7:**
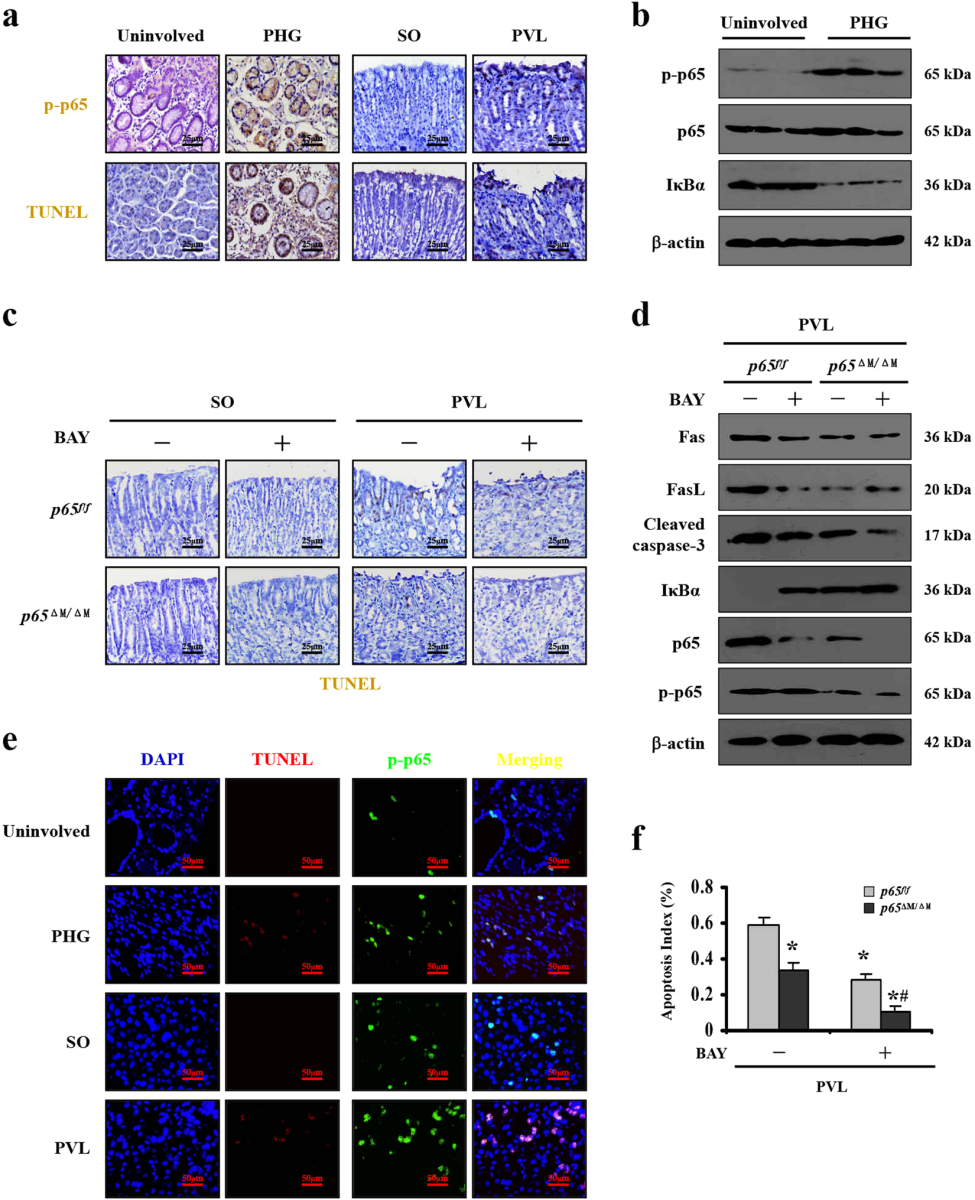
Fas/FasL promoted epithelial apoptosis via NF-κBp65 in PHG. **a** Representative images of NF-κBp-p65 and TUNEL staining from the indicated tissues (brown, × 400, *n* = 6 per group). **b** Western blotting represented that NF-κBp-p65 was upregulated in gastropathic mucosal tissue from PHG patients. β-actin was used as the loading control. *n* = 6 per group. **c** The NF-κB inhibitor BAY inhibited mucosal apoptosis both in PVL-treated *p65*^*f/f*^ and *p65*^ΔM/ΔM^ mice, without affecting that in SO (sham operation) mice (TUNEL staining, brown, × 400, *n* = 6 per group). **d** BAY inhibited caspase-3 cleavage, Fas, FasL, mucosal apoptosis and NF-κBp65 phosphorylation in PVL-treated *p65*^*f/f*^ mice. β-actin was used as the loading control. *n* = 6 per group. **e** Double staining of NF-κBp-p65 (green) and TUNEL (red), nuclei (blue) were counterstained with DAPI (× 800, *n* = 6 per group). **f** The apoptosis index was determined from (**c**) (upper panel). *n* = 6 in each group, values are presented as mean ± SEM. *P < 0.05 versus *p65*^*f/f*^ mice without BAY administration, #*P* < 0.05 versus *p65*^ΔM/ΔM^ mice without BAY administration

Our preceding data have testified that NF-κBp65 activation involved in gastric epithelial apoptosis in PHG^8^. Histological and protein analyses showed that NF-κBp65 phosphorylation (NF-κBp-p65), accompanied with obvious epithelial apoptosis, were increased in the PHG sections of both patients and mice (Fig. 7a, b). BAY, which could inhibit the activation of NF-κBp65, blocked the mucosal apoptosis both in PVL-treated *p65*^*f/f*^ and *p65*^ΔM/ΔM^ mice (Fig. 7c, f). However, BAY inhibited the cleavage of caspase-3 and mucosal apoptosis, and had no obvious effect on either Fas or FasL repression in PVL-treated *p65*^ΔM/ΔM^ mice, although it did alleviate Fas and FasL expression in PVL-treated *p65*^*f/f*^ mice (Fig. 7d). Double staining suggested NF-κBp-p65 and Fas signaling were located in the similar cells (Supplementary Fig. 3a), and co-staining of Fas and epithelial marker cytokeratin were mainly co-located (Supplementary Fig. 3b). Further, marked gastric apoptosis and higher p-p65 expression were synchronously observed in PHG patients and mice (Fig. 7e). These data demonstrated NF-κBp65 in myeloid cells promoted FasL production, while in epithelial cells, it enhanced Fas-mediated mucosal apoptosis.

### PUMA participated in Fas/FasL/NF-κBp65-mediated epithelial apoptosis in PHG

**Fig. 8:**
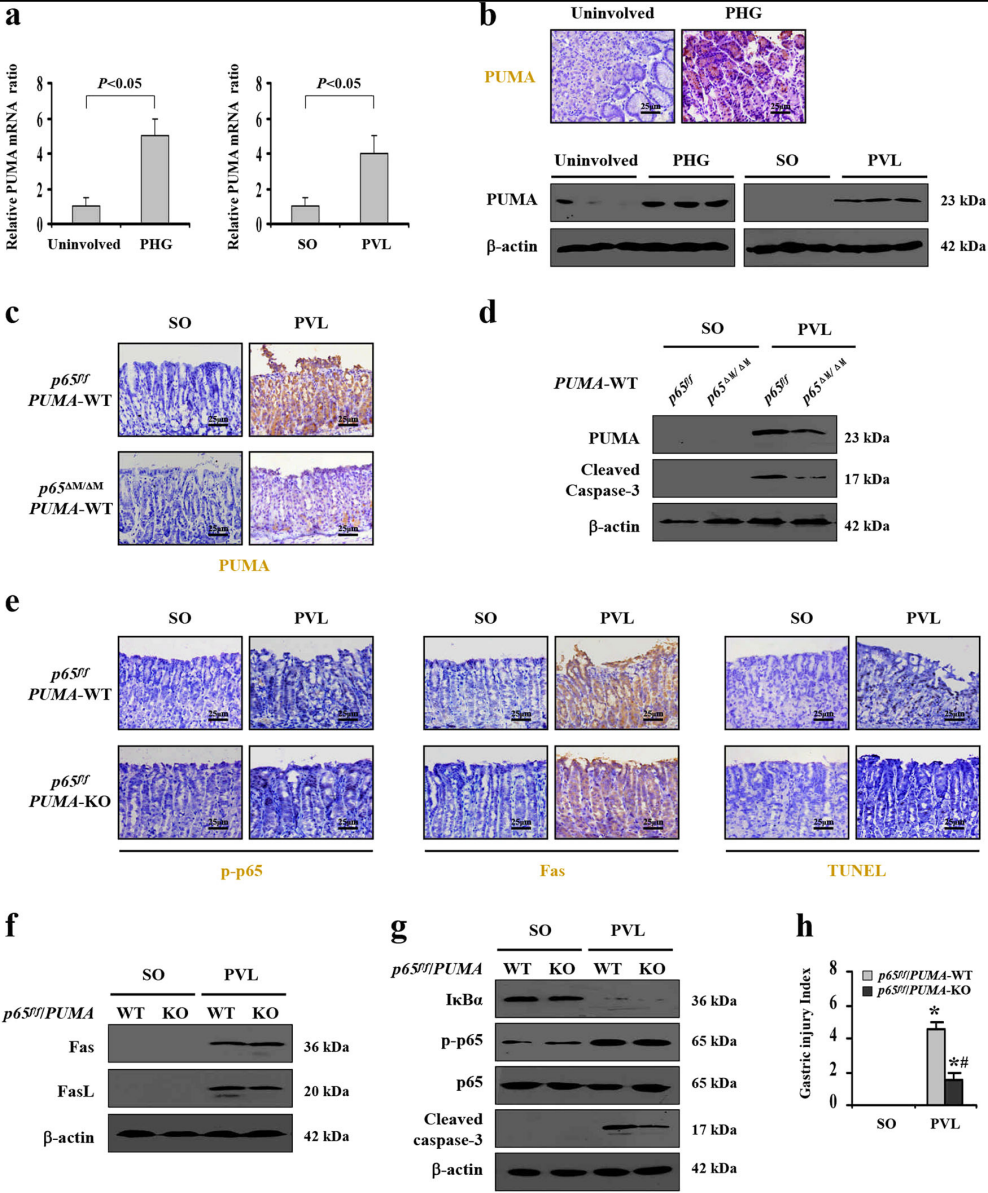
PUMA participated in Fas/FasL/NF-κBp65-mediated epithelial apoptosis in PHG. **a** Real-time PCR suggested *PUMA* mRNA expression was increased by fivefold in PHG and fourfold in PVL-treated mice, compared with their control group, respectively. *β-actin* was used as an internal control. *n* = 6 in each group, values are presented as mean ± SEM. Bonferroni’s comparison post hoc test. **b** Immunohistochemical staining of PUMA was presented (brown, upper panel, × 400, *n* = 6 per group). The levels of PUMA in the indicated tissues were analyzed by western blotting (lower panel). β-actin was used as the loading control. **c** PUMA staining demonstrated that deletion of *NF-κBp65* in myeloid cells of *PUMA*-WT mice repressed the expression of PUMA (brown, × 400, *n* *=* 6 per group). **d** Western blotting indicated that deletion of *NF-κBp65* in myeloid cells of *PUMA*-WT mice attenuated PUMA-mediated apoptosis (left panel). β-actin was used as the loading control. *n* = 6 per group. **e** Immunohistochemical staining showed deletion of *PUMA* in *p65*^*f/f*^ mice attenuated PVL-induced apoptosis (TUNEL staining) without affecting the station of NF-κBp65 activity (p-p65 staining) and Fas level (brown, × 400, *n* = 6 per group). **f**, **g** Western blotting depicted that *PUMA* deficiency in *p65*^*f/f*^ mice suppressed cleaved caspase-3 expression without influencing the levels of Fas, FasL and NF-κBp65 activity in PVL-treated mice. β-actin was used as the loading control. *n* = 6 per group. **h** The gastric injury index analysis represented that gastric mucosal injury was attenuated in PVL-treated *p65*^*f/f*^/*PUMA*-KO mice compared with *p65*^*f/f*^/*PUMA*-WT mice. *n* = 6 in each group, values are presented as mean ± SEM. *P < 0.05 versus SO mice, ^#^*P* < 0.05 versus PVL-treated *p65*^*f/f*^/*PUMA*-WT mice

**Fig. 9:**
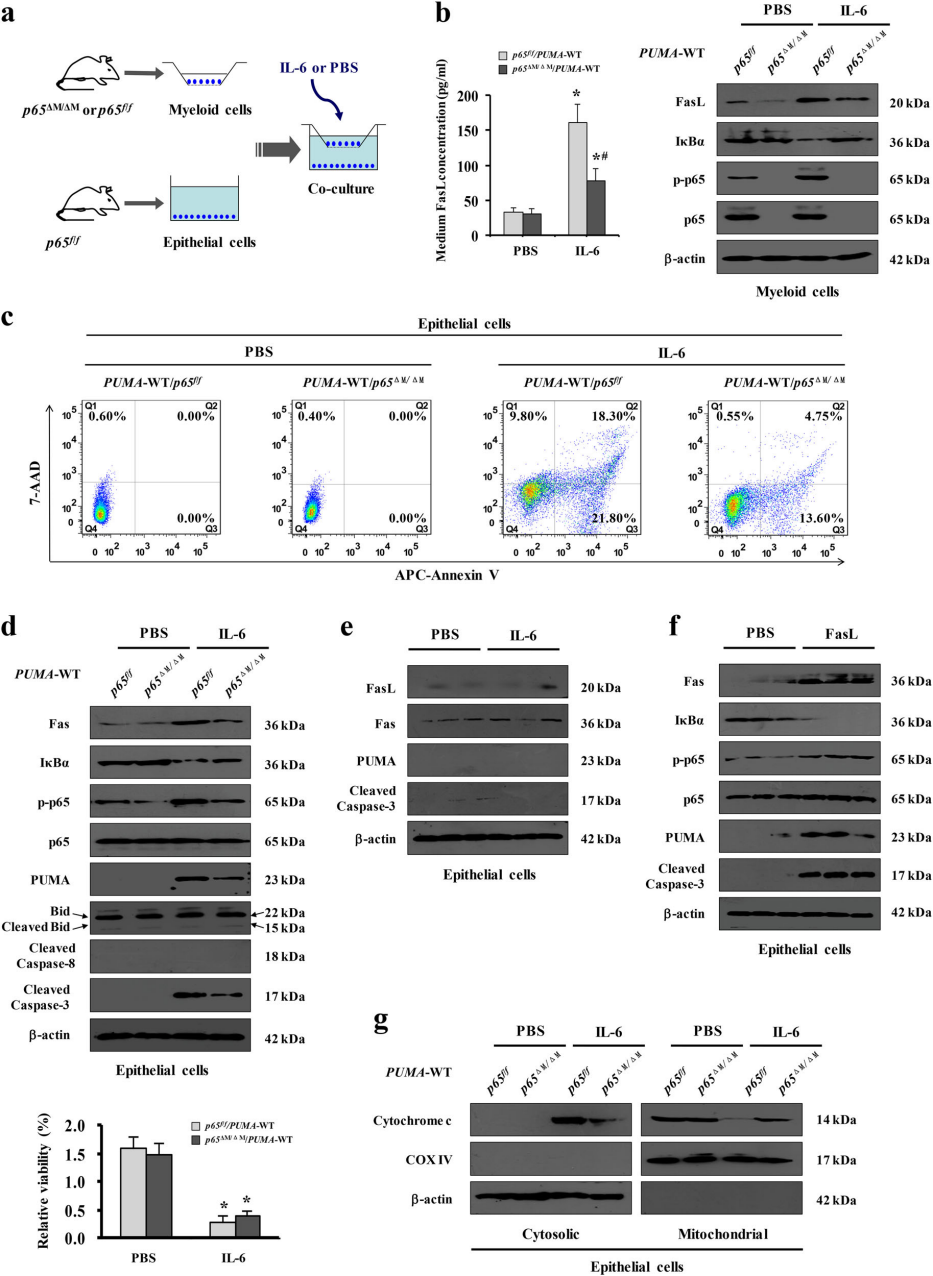
IL-6-driven FasL in myeloid cells facilitated PUMA-mediated epithelial apoptosis in vitro. **a** Schematic diagram of the co-culture experiments with primary myeloid cells and epithelial cells isolated from mice. **b** The concentration of FasL in the indicated medium of (**a**) was detected with ELISA (left panel, *n* = 6 per group). Values are presented as mean ± SEM. *P < 0.05 versus the medium of PBS-treated cells, ^#^*P* < 0.05 versus the medium of IL-6-treated *p65*^*f/f*^/*PUMA*-WT cells. Expressions of the related proteins of primary myeloid cells were detected by western blotting (right panel, *n* = 6 per group). **c** The extent of primary epithelial cells apoptosis from the co-culture experiments was preformed using an Annexin V-PE/7-AAD staining kit (Flow cytometric analysis). **d** The indicated proteins from primary epithelial cells in co-culture experiments were presented, and deletion of *NF-κBp65* in myeloid cells of *PUMA*-WT mice repressed Fas, NF-κBp-p65, PUMA and cleaved caspase-3, without affecting Bid and caspase-8 cleavage, in epithelial cells under IL-6 treatment in co-culture (upper panel). The epithelial cells viability was analyzed by CCK-8 assay (lower panel). *P < 0.05 versus the medium of PBS-treated cells. **e** Western blotting depicted IL-6 treatment did not influenced the levels of Fas, FasL, PUMA and cleaved caspase-3 of the isolated primary epithelial cells. β-actin was used as the loading control. *n* = 6 per group. **f** FasL treatment enhanced the expressions of Fas, NF-κBp-p65, PUMA and cleaved caspase-3 of the isolated primary epithelial cells. β-actin was used as the loading control. *n* = 6 per group. **g** Mitochondrial a*n*d cytosolic fractions were analyzed for cytochrome c by western blotting (*n* = 6 per group). β-actin and COX IV were used as the loading control of cytosolic and mitochondrial fractions, respectively

PUMA has been demonstrated to be a novel target of NF-κBp65 and a critical mediator of gastric epithelial apoptosis^19,24,25^. The enhanced expression of PUMA mRNA and protein was obviously presented in both PHG patients and mice compared with their normal groups (Fig. 8a, b). Deletion of *NF-κBp65* in myeloid cells of *PUMA*-WT mice from the PVL-treated group repressed the expression of PUMA, and few PUMA positive cells were detected in the gastric mucosa in SO mice from both the *p65*^*f/f*^/*PUMA*-WT mice and *p65*^ΔM/ΔM^/*PUMA*-WT mice (Fig. 8c). The cleavage of caspase-3 was repressed in PVL-treated *p65*^ΔM/ΔM^/*PUMA*-WT mice (Fig. 8d). *PUMA*-KO mice from *p65*^*f/f*^ littermates were utilized, and we found that deletion of *PUMA* in *p65*^*f/f*^ mice attenuated PVL-induced epithelial apoptosis and gastric injury without affecting NF-κBp65 activity and Fas levels (Fig. 8e, h). *PUMA* deficiency in *p65*^*f/f*^ mice suppressed pro-apoptotic protein caspase-3 activation without influencing the levels of Fas, FasL and NF-κBp65 activity in PHG mice (Fig. 8f, g), suggesting that NF-κBp65 is a crucial upstream regulator of PUMA that contributes to Fas/FasL-mediated epithelial apoptosis in PHG. By utilizing co-culture experiments with primary myeloid cells and epithelial cells isolated from mice (Fig. 9a), we found that the concentration of FasL in the medium of the co-culture system was increased under IL-6 treatment, while deletion of *NF-κBp65* in myeloid cells of *PUMA*-WT mice repressed that increase (Fig. 9b). IL-6 enhanced the activation of NF-κBp65 and FasL levels in myeloid cells, while targeted deletion of *NF-κBp65* in myeloid cells of *PUMA*-WT mice abolished that above-mentioned responses (Fig. 9b). Flow cytometric analysis revealed the early and late apoptosis of primary epithelial cells in the co-culture system under IL-6 treatment was obviously increased compared with their control group, while deletion of *NF-κBp65* in myeloid cells in the co-culture system under IL-6 treatment repressed the percentage of early and late epithelial apoptosis (the early apoptotic cells: 13.60% versus 21.80%; the late apoptotic cells: 4.75% versus 18.3%) (Fig. 9c). The levels of Fas, NF-κBp-p65, PUMA and cleaved caspase-3 rather than cleaved Bid and cleaved caspase-8 in primary epithelial cells in the co-culture system were upregulated under IL-6 treatment, and deletion of *NF-κBp65* in myeloid cells of *PUMA*-WT mice repressed the changes in epithelial cells (Fig. 9d). CCK-8 assay revealed that the epithelial cells viability in the system was markedly repressed under IL-6 treatment, regardless of the deletion of *NF-κBp65* in myeloid cells or not (Fig. 9d). These data suggested that IL-6 drove FasL via NF-κBp65 in myeloid cells, which could facilitate NF-κBp65/PUMA-mediated epithelial apoptosis and repress cells viability in vitro. We found that IL-6 treatment could not directly regulate the levels of Fas, FasL, PUMA and cleaved caspase-3 in the primary epithelial cells dissociated from normal mice, while FasL administration promoted the expressions of Fas, NF-κBp-p65, PUMA and cleaved caspase-3 of the primary epithelial cells (Fig. 9e, f). To investigate the mechanisms of PUMA-mediated intrinsic apoptosis, mitochondrial and cytosolic fractions were purified through differential centrifugation from the primary epithelial cells in the co-culture system. The release of cytochrome c was examined by western blotting. In the co-culture system under PBS treatment, cytochrome c was detected in the mitochondrial fractions but not in the cytosolic fractions, while IL-6 treatment increased the amount of cytochrome c in the cytosolic fractions of the epithelial cells in the co-culture system, especially in the epithelial cells co-cultured with the myeloid cells from *p65*^*f/f*^/*PUMA*-WT mice (Fig. 9g). In summary, PUMA was a critical mediator of Fas/FasL/NF-κBp65-mediated epithelial apoptosis in PHG.

## Discussion

The gastric mucosa from PHG showed a significant destruction of architecture, edema with erosion, vasodilatation with inflammatory cell infiltration and obvious epithelial apoptosis induction compared with the uninvolved normal mucosa^2,3,6^. Our previous studies have demonstrated that TNF-α/TNFR1 and FasL/Fas contributed to gastric mucosal apoptosis via increasing the activity of caspase family members, and inflammatory cells-driven TNF-α played a vital role in ER stress/PUMA-mediated mucosal epithelial apoptosis via regulating NF-κBp65/inducible nitric oxide synthase (iNOS)/NO signaling in PHG^3,7^. However, the in-depth mechanism of Fas/FasL activation in PHG remains largely unknown. In the current study, we showed that IL-6 drove FasL production via NF-κBp65 in myeloid cells to promote NF-κBp65/PUMA-mediated mucosal injury and epithelial apoptosis via Fas receptor in PHG, and inhibition of IL-6 networks or deletion of *NF-κBp65* in myeloid cells alleviated the inflammatory response and Fas/FasL-mediated epithelial apoptosis in PHG, suggesting that, unlike TNF-α networks, IL-6-driven Fas/FasL elements played a specific role in the mucosal injury and apoptosis of PHG.

The pathogenesis of PHG is complex, and many controversies exist. Splanchnic blood flow, distribution of gastric blood, various factors, local disturbances and portal pressure have been examined to elucidate the underlying mechanisms^26^. In addition to vascular alterations and gastric blood flow change, histological evidence of non-specific inflammation has been described in PHG, and around the blood vessels in the conjunctive of lamina propria appeared frequently inflammatory cell infiltrations and enhanced cytokine production appeared frequently^27,28^. By detecting the mucosa of the PVL-induced PHG mouse and patient samples, we found that, accompanying with inflammation infiltration, the levels of *IL-1α*, *IL-1β*, *TNF-α*, *TGF-β*, *IL-6*, and *ICAM-1* were markedly increased in PHG tissues. Among these, IL-6 was expressed at the highest level and was located in nearly all the gastric mesenchymal cells. Furthermore, the selective IL-6 receptor (IL-6R) inhibitor Tocilizumab alleviated macrophage, B cell, T cell and neutrophil infiltration in PHG mice. Moreover, blockage of IL-6 suppressed Fas/FasL levels without affecting TNF-α/TNFR1 and TRAIL/DR4/DR5 induction, although it did alleviate mucosal epithelial apoptosis in PVL-treated mice, indicating that IL-6-driven mucosal epithelial apoptosis depended on the Fas/FasL signaling rather than the TNF-α/TNFR1 and TRAIL/DR4/DR5 networks.

IL-6 is produced by and elicits responses in a wide range of target cells and regulates immunity through effects on the generation, recruitment and functional phenotype of neutrophils, macrophages, and dendritic cells via IL-6R^21,29^. IL-6, as well as other inflammatory cytokines such as TNF-α and IL-1β, has been postulated to contribute to the production of numerous proteins, such as C-reactive protein (CRP), serum amyloid A (SAA), fibrinogen, VEGF, and glucagon-like peptide-1 (GLP-1), in various cells, These proteins participate in immune cells via activation of the NF-κB or STAT3 pathways, which lead to the onset or development of various diseases^21,30^. By analyzing the primary epithelial and myeloid cells isolated from mice, we found that IL-6 induced FasL almost in myeloid cells via NF-κBp65 activation rather than STAT3 and ERK1/2 elements, which could further stimulate its receptor Fas upregulation in epithelial cells. Furthermore, deletion of *NF-κBp65* in myeloid cells ameliorated FasL deposition and Fas-mediated epithelial apoptosis in PHG mice. While, IL-6 could not directly mediate epithelial cell Fas induction and apoptosis by utilizing primary epithelial cells and the gastric epithelial cell lines, GES-1, although it was reported that IL-6 involved in regulating apoptosis resistance in immune and non-immune cells, such as hepatocytes, myeloma cells, and colon carcinoma cells^31,32^, which may be related to the different cell types, tissue microenvironments and receptor/signaling system of diverse disorder states, which indeed merits further evaluation.

Our previous and current data elucidated that Fas/FasL levels, in parallel with the increased apoptotic epithelial cells, were induced in the mucosa of PHG^7^. Apoptosis is executed by the engagement and coaggregation of FasL with its receptor Fas on the cell surface followed by various intracellular elements interactions that coordinate the hierarchical activation of caspases and cell death^33^. Isolated primary cells and co-staining analysis demonstrated Fas was mainly localized in the mucosal epithelial cells and contributed to their apoptosis happening. Interestingly, *NF-κBp65* deficiency in gastric myeloid cells inhibited FasL expression, followed by Fas downregulation, revealing that IL-6-driven FasL in myeloid cells contributed to the epithelial apoptosis via Fas in PHG, which is consistent with the observation that immune cells could facilitate the integration of the pathophysiological mechanisms involved in the different complications of portal hypertension, such as PHG.

As the main functional element of NF-κB family, NF-κBp65 was revealed to be responsible for mediating TNF-α-driven epithelial apoptosis through ER stress/PUMA pathways in PHG and TNF-α/PUMA-induced apoptosis in a variety of tissues and cell types, including small intestinal epithelial cells, hepatocytes, and thymocytes^19^. To test whether this condition could also occur in Fas/FasL-mediated PHG, the NF-κBp65 inhibitor Bay11708 (BAY) was adopted, BAY inhibited mucosal apoptosis but had no repressing effect on both Fas and FasL in *p65*^ΔM/ΔM^ PHG mice, although it did alleviate Fas and FasL expression in PVL-treated *p65*^*f/f*^ mice, indicating that NF-κBp65 played a disparate role in gastric myeloid cells and epithelial cells of PHG, that was, promoting FasL production in myeloid cells and enhancing apoptosis in epithelial cells. We and others identified PUMA, as a novel target of NF-κBp65, and showed that it played an essential role in mediating cell apoptosis in vitro and in vivo^8,34^. Upon activation by NF-κBp65 through cell death receptors such as TNF-α or Fas/FasL, PUMA promotes caspases activation to trigger cells death^19,35^. Knockout of *PUMA* was testified to dramatically prevent ischemia/reperfusion-induced intestinal apoptosis, portal hypertension-induced gastric apoptosis and inflammation-driven colonic apoptosis, etc^8,36,37^. In the present study, we found that deletion of myeloid cells *NF-κBp65* repressed the expression of PUMA and PUMA-mediated apoptosis in PHG. Moreover, PUMA, as the downstream target of NF-κBp65, evidently enhanced epithelial injury and apoptosis induced by Fas/FasL/NF-κBp65 networks in PHG. These findings further broaden the regulatory network of epithelial apoptosis and deepen our understanding of the molecular mechanism of PHG.

In summary, IL-6 drives FasL production via NF-κBp65 in myeloid cells to promote Fas/NF-κBp65/PUMA-mediated epithelial apoptosis in PHG, and this network provides a potential therapeutic target for PHG.

## Materials and methods

### Tissue samples

The gastric mucosal specimens of 15 PHG patients (female/male: 8/7; ages: 49.47 ± 7.33. Among them, 8 with hepatitis B virus (HBV)-infected liver cirrhosis and 7 with portal vein occlusion (from cavernous transformation of the portal vein or portal vein thrombosis without cirrhosis)) without *helicobacter pylori* infection before any therapeutic intervention and 15 uninvolved healthy volunteers (female/male: 7/8; ages: 50.00 ± 5.12) from the people of a regular healthy physical examination were obtained from The Endoscopic Center of The Third Affiliated Hospital of Sun Yat-Sen University, and the characteristics of the uninvolved healthy volunteers and PHG patients are shown in Supplementary Table 1. The PHG patients and healthy volunteers were matched in terms of gender and ages (gender: *P* = 1.000; ages: 49.47 ± 7.33 vs 50.00 ± 5.12, *P* = 0.819). Due to the most common locations for PHG are the corpus and fundus of the stomach, and the fundus of PHG is always associated with gastric varices^1,2,4^, the gastric specimens of healthy volunteers and PHG patients were all come from the corpus of the stomach. Written informed consent was acquired from each patient and healthy volunteer before inclusion in the study and the endoscopic inspectors were blinded toward the groups before the endoscopic examinations. The study protocol and the acquisition of the tissue samples were approved by the Institutional Review Board of The Third Affiliated Hospital of Sun Yat-Sen University.

### Mice and induction of portal hypertension

All animal experiments were approved by the Institutional Animal Care and Use Committee at Sun Yat-Sen University. LoxP *NF-κBp65* (*RelA, p65*^*flox/flox*^) mice were generated on a C57BL/6 gene background. Mice expressing the lysozyme promoter-driven *cre* recombinase gene (*LysM-cre* mice) on the C57BL/6 genetic background were kindly provided by Dr. Jianping Ye (Pennington Biomedical Research Center, Louisiana State University System, Baton Rouge, LA, USA). Myeloid cells specific *NF-κBp65* deletion mice (*p65*^ΔM/ΔM^) were generated by crossing the floxed *p65* mice with *LysM-cre* mice, which show *NF-κBp65* ablated solely in myeloid cells, including monocytes, mature macrophages, and granulocytes. Floxed *p65* littermates (*p65*^*flox/flox*^, also as *p65*^*f/f*^) were used as the wild-type (WT) mice. *PUMA*^*+/–*^ heterozygous mice (Jackson Laboratory, Bar Harbor, Maine, USA) in the C57BL/6 background were crossed with *p65*^ΔM/ΔM^ or *p65*^*f/f*^ to generate *p65*^ΔM/ΔM^*/PUMA*-WT, *p65*^ΔM/ΔM^*/PUMA*-KO, *p65*^*f/f*^*/PUMA*-WT, and *p65*^*f/f*^*/PUMA*-KO littermates, respectively. Mice were housed in micro-isolator cages in a room illuminated from 7:00 am to 7:00 pm (12:12 h light: dark cycle; a climatically controlled environment (22 ± 2 °C)) and were allowed access to water and chow ad libitum. All experiments were conducted in a blinded manner and the mice were randomly allocated to the indicated groups. All mice were killed by excessive carbon dioxide inhalation at the end of the experiments.

Mice portal hypertension gastropathy was induced by a portal vein ligation (PVL) technique as previously described^3,8^. The portal vein was carefully isolated and calibrated constriction was performed using a single ligature of 3-0 silk around the portal vein and a 20-gauge blunt-tipped needle under anesthetized by Ketamine & Xylazine mixed anesthetic. The needle was then removed, leaving a calibrated stenosis of ~50% of its diameter of the initial portal vein. In sham operation (SO) mice, the same operation was performed without ligation after isolating the portal vein. After the above operation, all animals were housed in cages and allowed free access to food and water for 2 weeks. In some experiments, mice after operation were injected with vehicle (as control) or the selective IL-6 receptor inhibitor Tocilizumab (6 mg/kg, Calbiochem, La Jolla, CA, USA) every other day for 2 weeks or 200 μg/per mouse Bay11708 (BAY, the NF-κB inhibitor, Calbiochem) daily for 2 weeks, respectively. For IL-6 treatment experiments, mice were injected with vehicle (PBS) or recombinant murine IL-6 (80 ng/per mouse, R&D Systems, Minneapolis, MN, USA), respectively. Mice were killed at 8 h after PBS or IL-6 injection.

### Samples collection

Immediately after the animals were killed, the entire stomach was carefully removed and rinsed thoroughly with ice-cold physiological saline and then opened to expose the gastric mucosa as previously described^3,8^. The mucosal layers were harvested and stored at −80 °C before protein and mRNA analyses. The entire stomach was fixed in neutral buffered formalin to prepare paraffin sections. Gastric specimens of healthy volunteers and PHG patients were processed by the similar way.

### Histological and TUNEL staining

Terminal deoxynucleotidyl transferase-mediated deoxyuridine triphosphate nick-end labeling (TUNEL) was performed by using the detection kit according to the manufacturer’s instructions. The apoptotic index was determined by dividing the number of apoptotic cells by the total number of cells in at least 20 randomly selected fields (×200)^3,8,38^. The entire stomach was carefully removed and rinsed thoroughly and then opened on its lesser curvature longitudinally to expose the gastric mucosa, and gastric injury index was analyzed blindly based on the previously described criteria^3^: 0: normal; 1: mucosa with erosion; 2: mucosa with ulcer (<1 mm); 3: mucosa with ulcer (1–2 mm); 4: mucosa with ulcer (3–4 mm); 5: mucosa with ulcer (>5 mm).

### Immunohistochemical and immunofluorescence staining

For immunohistochemical (IHC) staining, the slides were deparaffinized, rehydrated and treated with antigen retrieval, and then the slides were incubated with the indicated antibody. An HRP-conjugated antibody was used as the secondary antibody and followed by detection using the ABC staining system, and finally the sections were counterstained with hematoxylin. For immunofluorescence (IF) staining, antibody-antigen complexes were visualized by incubation with biotin-conjugated secondary antibody and Streptavidin Alexa 488 (Invitrogen, Carlsbad, CA, USA) or 594 (Molecular Probes, Eugene, OR, USA), with nuclei counterstained with 2 mg/ml of 4′,6-diamidino-2-phenylindole dihydrochloride (DAPI, Molecular Probes). For co-staining, the slides were adopted to detect the secondary targeted protein after finishing the first protein detection. For staining in tissues, the slides were incubated with primary antibodies against IL-6, CD68 and MPO (all from Santa Cruz, Santa Cruz, CA, USA), cytokertin and PUMA (all from Abcam, Cambridge, MA, USA), and CD3, CD19, NF-κBp-p65 (all from Cell Signaling Technology, Danvers, MA, USA), and Fas, FasL (all from Sigma, St Louis, MO, USA).

### Analysis of cytochrome c release

To analyze the release of cytochrome c, an aliquot of each epithelial sample was used to isolate mitochondrial and cytosolic fractions by the differential centrifugation method as previously described^38,39^.

### Western blotting

Total proteins were prepared from the related freshly isolated tissues, and were then homogenized in the western blotting analysis buffer (10 mM Tris-HCl (pH 7.4), 150 mM NaCl, 1% Triton X-100, 1% sodium deoxycholate, 0.1% sodium dodecyl sulfate (SDS), 5 mM ethylenediaminetetraacetic acid (EDTA), 1 mM phenylmethanesulfonyl fluoride (PMSF), 0.28 kU/L aprotinin, 50 mg/L leupeptin, 1 mM benzamidine, and 7 mg/L pepstain A) to prepare the homogenate. The homogenate was then centrifuged at 10,000 × *g* for 20 min at 4 °C, and the supernatant was collected as the protein lysate and preserved at −80 °C for later analysis. After quantification, equal amounts of protein lysate from each sample was subject to electrophoresis on 10% sodium dodecyl sulfate-polyacrylamide gel electrophoresis (SDS-PAGE) utilizing a constant current, and then transferred onto the polyvinylidene fluoride (PVDF) membranes (Millipore, Billerica, MA, USA) on a semidry electrotransferring unit. The membranes were blocked in 5% nonfat milk and incubated with anti-IL-6, -Fas, -FasL, -cleaved caspase-3 (Cell Signaling Technology), -cleaved caspase-8 (Cell Signaling Technology), -Bid (Cell Signaling Technology), -Cytochrome c (Cell Signaling Technology), -TNF-α (Cell Signaling Technology), -ERK1/2 and -p-ERK1/2 (Cell Signaling Technology), -TNFR1 (Abcam), -TRAIL (Abcam), -DR4 (Santa Cruz), -DR5 (Santa Cruz), -PUMA (Abcam), -IL-6R (Abcam), -NF-κBp-p65, -NF-κBp65 (Sigma), -IκBα (Abcam), -STAT3 (Sigma), -p-STAT3 (Sigma), -COX IV (Santa Cruz), or -β-actin (Sigma), respectively. After the overnight incubation with the above-mentioned antibodies, membranes were washed, and the appropriate horseradish peroxidase-conjugated secondary antibodies were used to detect the primary antibody/antigen complexes, and finally the signal was detected using ECL detection reagents (Amersham Pharmacia Biotech, Piscataway, NJ, USA)^3,38^.

### Real-time polymerase chain reaction (real-time PCR)

Total RNA was isolated and reversed transcribed with SuperScript III reverse transcriptase (Invitrogen, Carlsbad, CA, USA) according to the manufacturer’s instructions. The indicated mRNA expression was analyzed by real-time PCR that performed with a Mini Opticon Real-time PCR System (BioRad, Hercules, CA, USA) on a Chromo 4 Detector System (MJ Research, Sierra Point, CA, USA) using gene specific primers and DyNAmo SYBR Green Master Mix (Finnzymes, Finland), as previously described^3,38^. *IL-1α*, *IL-1β*, *TNF-α*, *TGF-β*, *IL-6*, *ICAM-1*, *Fas*, *Fasl*, and *PUMA* were amplified using primers (Invitrogen). As an internal control, the expression of *β-actin* in each sample was also quantified using the sense primers (Invitrogen). Relative quantitative algorithm (as relative mRNA ratio) was used to analyze the data. For controlling unwanted sources of variation, each sample was normalized by *β-actin* gene in the same sample. The formula was the relative expression of genes = 2^−ΔΔCt^, and ΔΔCt value = [(target gene Ct value in the treatment group − β-actin Ct value in the treatment group) − (target gene Ct value in the control group − β-actin Ct value in the control group)]. *n* = 6 in each group, values are presented as mean ± SEM (standard error of the mean).

### Primary cells isolation and cells culture

Primary gastric cells were dissociated from the indicated mice by a non-recirculating collagenase perfusion as previously described^3^. The entire stomach was carefully removed and rinsed, ligated the pylorus and then was perfused through the cardia with Ca^2+^-free HBSS (Hank’s Balanced Salt solution) for 15 min, with 100 ml 0.2% pronase solution and with 0.2% collagenase Type-IV (Sigma) solution. The mucosal layers were harvested for cells suspension, and then filtered through 100 μm pore size mesh nylon filter (Sinopharm Chemical Reagent, Shanghai, China). The cells were centrifuged at 300 × *g* for 15 min, the pellet was collected for gastric epithelial cells isolation and the supernate for primary myeloid cells. Commercially available 30% Percoll (Sigma) was prepared and the above-mentioned pellet was added to the upper layer of Percoll carefully, and then was centrifuged at 450 × *g* for 20 min to collect gastric epithelial cells. For primary myeloid cells isolation, the above supernate was centrifuged at 1400 × *g* for 30 min and the pellet was collected for myeloid cells. The primary cells were cultured in RPMI medium 1640 supplemented with 10% heat-inactivated fetal bovine serum (FBS), 100 units/ml penicillin and 100 μg/ml streptomycin in a humidified incubator at 37 °C with 5% CO_2_, and after being cultured for 48 h, the primary cells obtained the highest viability assayed by the Cell Counting Kit-8 (CCK-8) analysis (Dojindo, Kumamoto, Japan). The primary isolated cells viability was detected by CCK-8 assay following the manufacturer’s protocol. In brief, appropriate cells were seeded in 96-well plates for 24–96 h, and 10 μl of CCK-8 solution was added to the corresponding wells and incubated in a 5% CO_2_ incubator for 2 h, and then measured at a wavelength of 450 nm. The GES-1 and RAW 264.7 cell lines were obtained from the American Type Culture Collection (ATCC, Manassas, VA, USA). The GES-1 cells were cultured in RPMI medium 1640 and RAW 264.7 cells in DMEM medium.

For IL-6 or FasL treatment experiments in vitro, recombinant IL-6 (10 ng/ml) or FasL (10 ng/ml, Sigma) were added for 12 h, and these cells were harvested for further analysis. For co-culture experiments, after being isolated from the related mice, the primary myeloid cells and epithelial cells were plated in the co-culture system with RPMI medium 1640 for 48 h, and then 10 ng/ml recombinant IL-6 (R&D Systems) or the same volume of PBS (the control group) were added into the co-culture system for 12 h. For flow cytometric experiments, the isolated primary epithelial and myeloid cells were stained with commercially available anti-Fas or anti-FasL, respectively. The primary epithelial cells apoptosis was quantified using an Annexin V-PE/7-AAD (7-amino-actinomycin D) (KeyGEN BioTECH, China) staining kit. Flow cytometric analysis was performed with BD FACS Calibur or BD FACS Aria (Becton Dickinson, NJ, USA) according to the manufacturer’s instruction, and the data were analyzed using FCS Express software Flowjo 7.6. For FasL concentration detection in the co-culture system, the concentration of FasL in the indicated medium was detected with ELISA (Enzyme-linked immunosorbent assay) kits (CUSABIO, Wuhan Huamei Biotech, China) according to the manufacturer’s instruction.

### Microarray analysis

Microarray experiment was performed as previously described^40^. Total RNA from each sample was dissociated according to the instructions of TRIzol reagent (Invitrogen) and was purified by using a mirVana miRNA Isolation Kit (Ambion, Austin, TX, USA). The cDNA labeled with Cy3-dCTP was generated by using the Eberwine’s linear RNA amplification method and enzymatic reaction, and then the double-stranded cDNA (dsDNA) products were purified and eluted according to the instructions of the PCR NucleoSpin Extract II Kit. The eluted dsDNA was then vacuums evaporated to 16 ml and subjected to the 40 ml transcription reactions at 37 °C for 14 h according to the instructions of a T7 Enzyme Mix as previously described^40^. Klenow enzyme labeling tactics was adopted following by reverse transcription using CbcScript II reverse transcriptase. Finally, array hybridization was performed in an Agilent Hybridization oven overnight. After that, the GeneSpring software V12array was adopted to summarize and normalize data. The Adjust Data function of CLUSTER 3.0 software was utilized for Log2 transformation and hierarchical clustering of the data. Java Treeview (Stanford University School of Medicine, Stanford, CA, USA) was performed for Tree visualization.

### Statistical analysis

All the experiments were repeated at least five times with the similar results. The data were expressed as the mean ± SEM (standard error of the mean), and data statistical analysis were performed using Student’s two-tailed paired *t*-test or one-way ANOVA (more than two groups of data, single factor) or two-way ANOVA (more than two groups of data, two factors), followed by Bonferroni’s comparison post hoc test and post hoc tests are run only if *F* achieved *P* < 0.05 and there was no significant variance inhomogeneity. Differences were considered statistically significant if the probability of the difference occurring by chance was <5 in 100 (*P* < 0.05).

## Supplementary information

Supplementary Table

Supplementary Figures
