# Supplementary material for: IL-6-driven FasL promotes NF-κBp65/PUMA-mediated apoptosis in portal hypertensive gastropathy
Source: Cell Death Dis. 2019 Oct 3;10(10):748. doi: 10.1038/s41419-019-1954-x (PMC6776649; doi:10.1038/s41419-019-1954-x)
Supplement: Supplementary file 1 — Supplementary Table [file 41419_2019_1954_MOESM1_ESM.doc]

**Supplementary Table 1** Characteristics of Uninvolved healthy volunteers and PHG patients

| **Variable** | **Uninvolved** | **PHG** | **P value** |
| --- | --- | --- | --- |
| **Age (years)** | 50.00±5.12 | 49.47±7.33 | 0.819 |
| **Gender (F/M)** | 7/8 | 8/7 | 1.000 |
| **AST (U/L)** | 19.07±4.72 | 30.87±11.49 | 0.001 |
| **ALT (U/L)** | 16.06±6.25 | 26.00±8.98 | 0.002 |
| **RBC (10^12^/L)** | 4.62±0.46 | 3.81±0.58 | ＜0.001 |
| **WBC (10^9^/L)** | 5.61±1.06 | 4.15±0.99 | 0.001 |
| **PLT (10^9^/L)** | 243.60±53.12 | 96.40±41.09 | ＜0.001 |
| **ALB (g/L)** | 44.27±3.49 | 37.53±3.81 | ＜0.001 |

PHG, portal hypertensive gastropathy; ALT, alanine transaminase; AST, aspartate transaminase; RBC, red blood cell; WBC, white blood cell; PLT, platelet; ALB, albumin.
