# Supplementary material for: IL-6-driven FasL promotes NF-κBp65/PUMA-mediated apoptosis in portal hypertensive gastropathy
Source: Cell Death Dis. 2019 Oct 3;10(10):748. doi: 10.1038/s41419-019-1954-x (PMC6776649; doi:10.1038/s41419-019-1954-x)
Supplement: Supplementary file 2 — Supplementary Figures [file 41419_2019_1954_MOESM2_ESM.doc]

**Supplementary Figures**

**
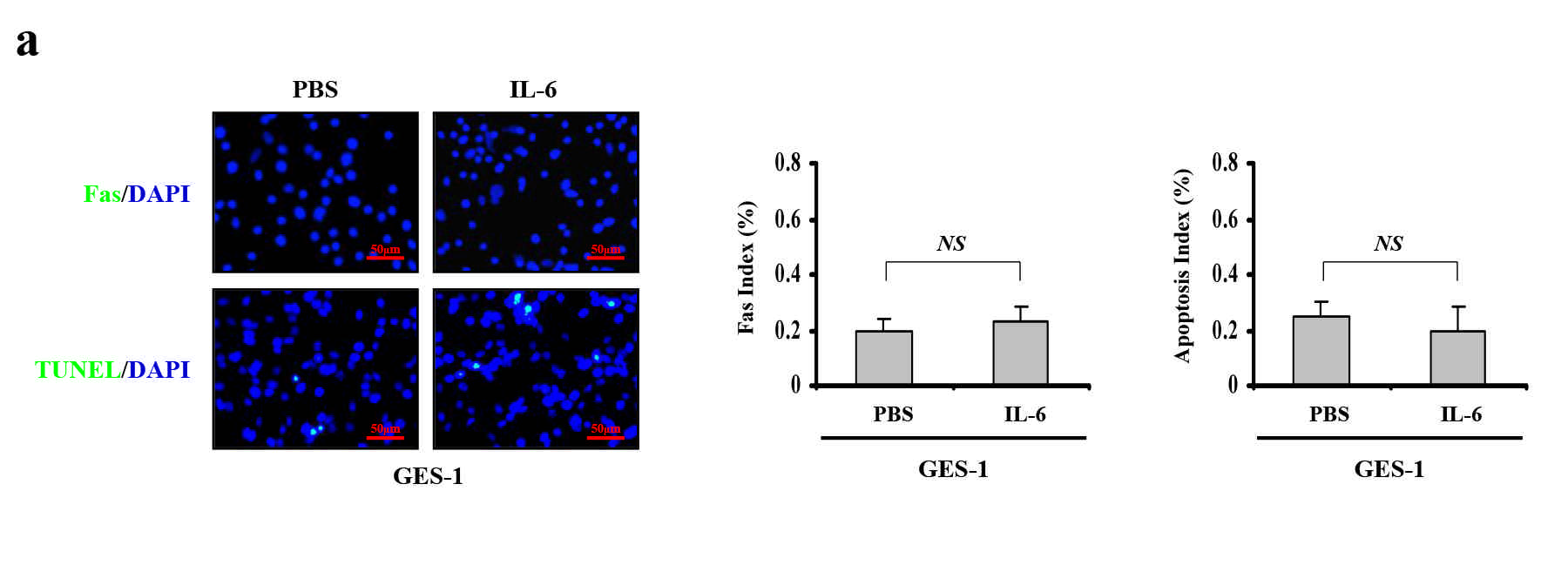
**

**Supplementary Fig. 1** IL-6 could not promote GES-1 apoptosis. **a** Immunofluorescence staining of Fas (green) and TUNEL staining (green) was presented. Cell nuclei (blue) were counterstained by DAPI (×800). The Fas index and the apoptotic index were also represented. *n* = 6 in each group, values are presented as mean ± SEM. *NS*, no significance.

**
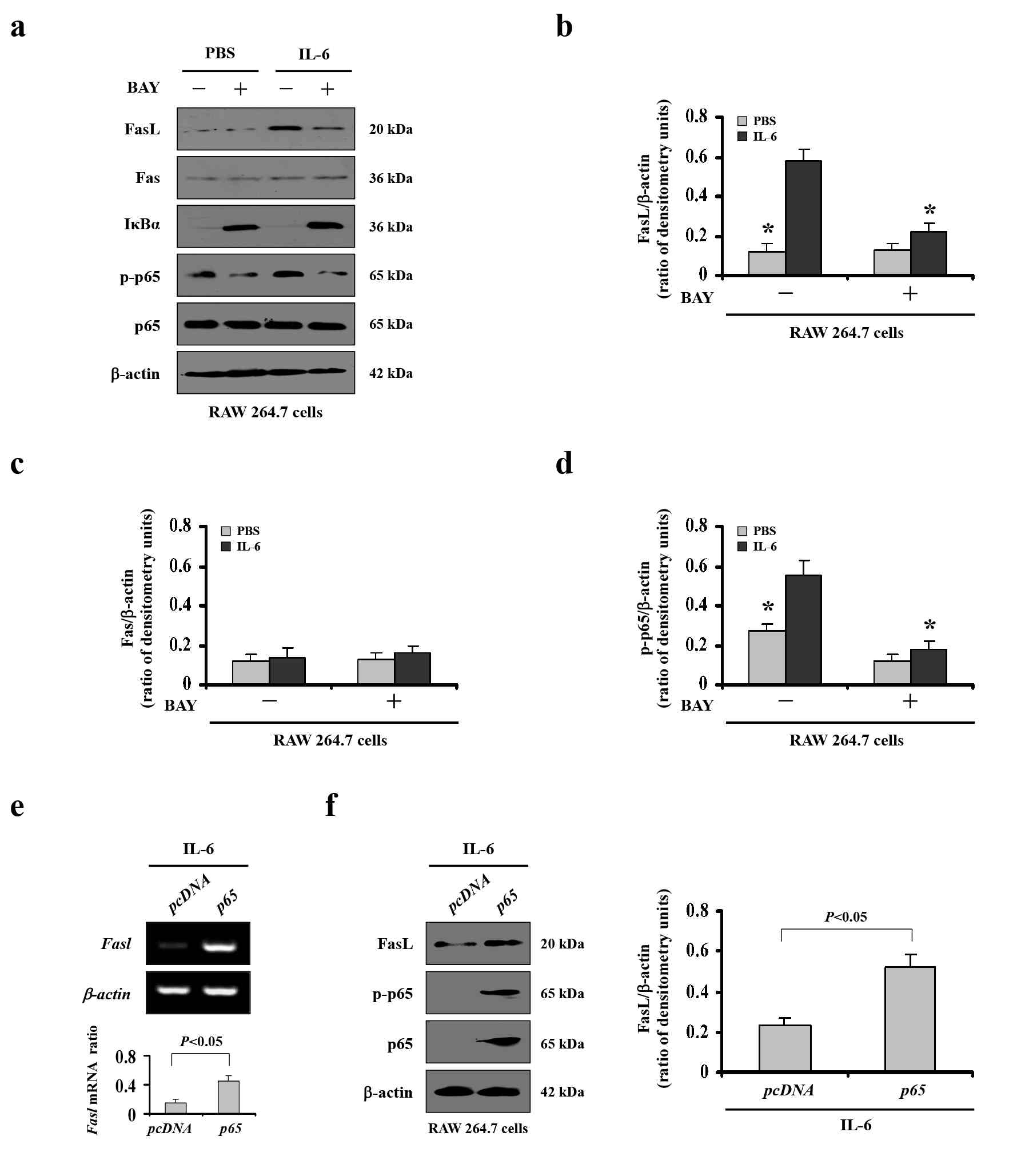
**

**Supplementary Fig. 2** IL-6 enhanced FasL production via NF-κBp65 in myeloid cells *in vitro*. **a** The NF-κB inhibitor Bay11708 (BAY) inhibited IL-6-induced FasL level and NF-κBp65 phosphorylation (NF-κBp-p65) in RAW 264.7 cells. β-actin was used as the loading control. *n* = 6 per group. **b-d** The ratio of densitometry units of FasL/β-actin, Fas/β-actin and NF-κBp-p65/β-actin was represented. *n* = 6 in each group, values are presented as mean ± SEM. **P*<0.05 versus IL-6-treated cells without BAY administration. **e** *Fasl* mRNA induction in RAW 264.7 cells by *NF-κBp65* transfection in IL-6 treatment (*n* = 6 per group). **f** The protein levels of NF-κBp-p65, NF-κBp65 and FasL in RAW 264.7 cells by *NF-κBp65* transfection and subsequent IL-6 treatment were determined by western blotting, and the ratio of FasL/β-actin was also presented. β-actin was used as the loading control. *n* = 6 per group, values are presented as mean ± SEM.

**
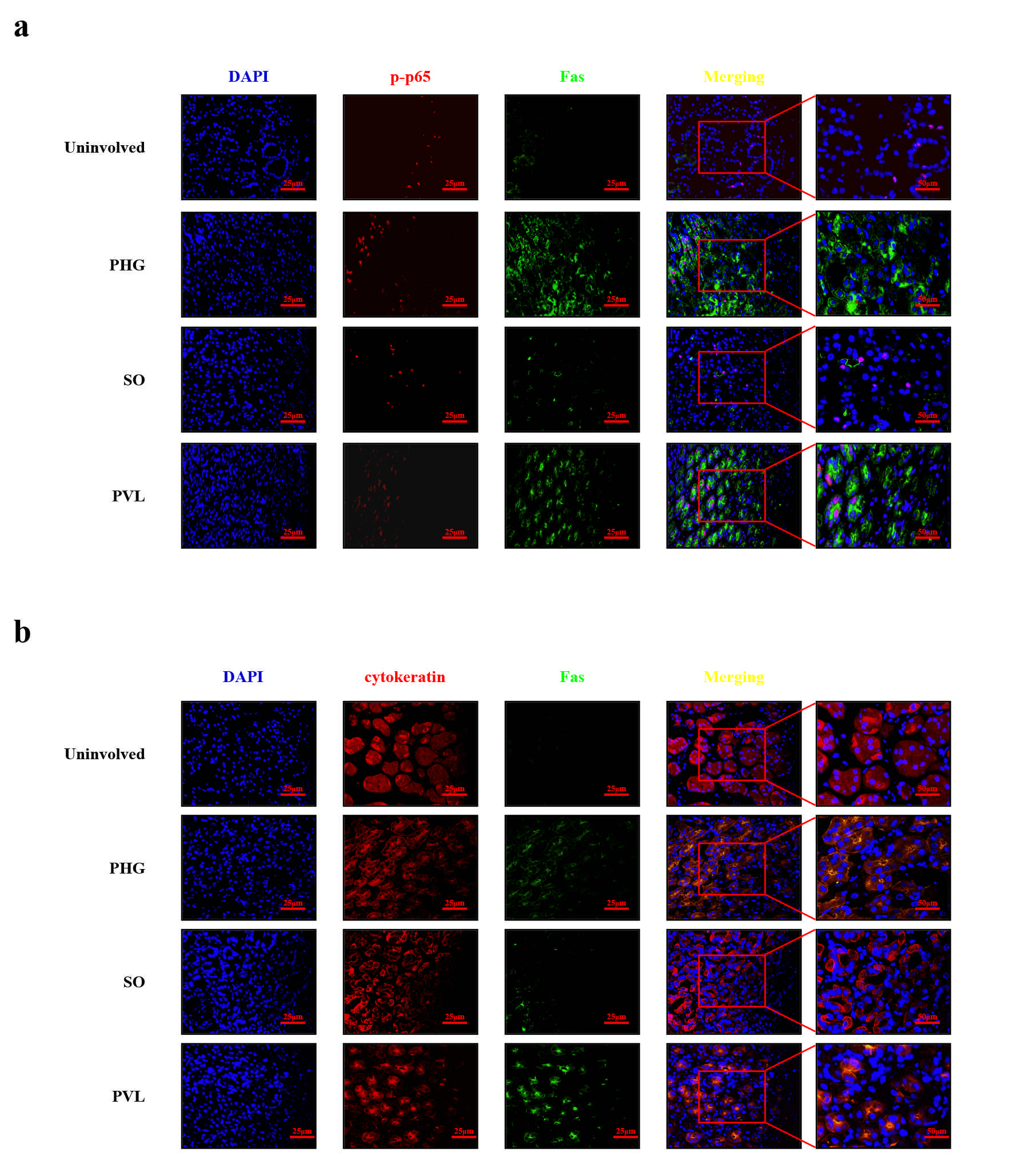
**

**Supplementary Fig. 3** Fas/FasL contributed to epithelial apoptosis via NF-κBp65 in PHG. **a** Double staining of NF-κBp-p65 (red) and Fas (green) indicated that NF-κBp65 activity and Fas signaling located in the similar mucosal cells, nuclei (blue) were counterstained with DAPI (×400, *n* = 6 per group). **b** Co-staining of cytokeratin (red) and Fas (green) demonstrated that Fas located in epithelial cells, nuclei (blue) were counterstained with DAPI (×400, *n* = 6 per group).
